# Alaska backcountry expeditionary hunting promotes rapid improvements in metabolic biomarkers in healthy males and females

**DOI:** 10.14814/phy2.14682

**Published:** 2020-12-28

**Authors:** Melynda S. Coker, Kaylee Ladd, Carl J. Murphy, Brent C. Ruby, Timothy C. Shriver, Dale A. Schoeller, Bradley R. Newcomer, Terry Bateman, Larry Bartlett, Robert H. Coker

**Affiliations:** ^1^ Department of Natural Resources and Environment University of Alaska Fairbanks Fairbanks AK USA; ^2^ Department of Biology and Wildlife University of Alaska Fairbanks Fairbanks AK USA; ^3^ Institute of Arctic Biology University of Alaska Fairbanks Fairbanks AK USA; ^4^ Montana Center for Work Physiology and Exercise Metabolism University of Montana Missoula MT USA; ^5^ Isotope Ratio Core Laboratory University of Wisconsin‐Madison Madison WI USA; ^6^ Department of Physics and Honors College James Madison University Harrisonburg VA USA; ^7^ Fairbanks Memorial Hospital Fairbanks AK USA; ^8^ Pristine Ventures, Inc Fairbanks AK USA

**Keywords:** adipose tissue, energy expenditure, intrahepatic lipid, serum lipid, skeletal muscle

## Abstract

We have previously reported negative energy balance and health benefits during an Alaska backcountry expeditionary hunting (ABEH) immersion in two males. The purpose of our present study was to increase the number of participants, include females, and evaluate macronutrient intake and serum lipids. Four men (age: 46 ± 6 year, BMI: 26 ± 1 kg/m^2^) and three women (age: 46 ± 11 year, BMI: 25 ± 3 kg/m^2^) were recruited. Doubly labeled water methodology and dietary recall were utilized to assess energy expenditure and energy intake, respectively. Data were collected during pre‐ and post‐ABEH visits. Body composition was measured using dual‐energy x‐ray absorptiometry and the cross‐sectional area of skeletal muscle in the upper leg (XT), and intrahepatic lipid (IHL) was determined using magnetic resonance imaging and/or spectroscopy (MRI/MRS). Blood parameters were measured by LabCorp. Paired T‐tests were used for statistical analysis. Data are reported as mean ± SD and considered significant at *p* < 0.05. Total energy intake was 7.7 ± 3.4 MJ/day and total energy expenditure was 17.4 ± 2.6 MJ/day, resulting in a negative energy balance of −9.7 ± 3.4 MJ/day. Protein intake(grams)/body weight(kilograms)/day was 1.0 ± 0.4. There were reductions in body weight (Δ‐1.5 ± 0.7 kg), BMI (Δ‐0.3 ± 0.2 kg/m^2^), fat mass (Δ‐1.7 ± 0.9 kg), and IHL (Δ‐0.3 ± 0.3% water peak). There were no changes in lean tissue mass (Δ0.6 ± 1.4 kg) or XT (Δ‐1.3 ± 3.3 cm^2^). There were significant reductions in total cholesterol (Δ‐44 ± 35 mg/dl), LDL‐cholesterol (Δ‐25 ± 14 mg/dl), VLDL‐cholesterol (Δ‐7 ± 7 mg/dl), and triglycerides (Δ‐35 ± 33 mg/dl). The ABEH immersion resulted in considerable negative energy balance and provided comprehensive benefits in metabolic health without any reduction in skeletal muscle.

## Graphical abstract

The majority of the scientific literature on exercise training has focused on standardized concepts. We assert that movement constancy of the hunter gatherer lifestyle may offer protection against metabolic abnormalities but has been largely understudied. In the context of unscripted negative energy balance, muscle preservation, and rapid improvements in metabolic biomarkers were demonstrated in both healthy females and males during an Alaskan backcountry hunting expedition.

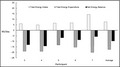

## 1 INTRODUCTION

The complex etiology of obesity and metabolic diseases on morbidity and mortality in the United States has been an intense point of investigation over the last century (Akram et al., 2000). Cumulative influential factors consistent with the agricultural, industrial, technological, and digital revolutions have led to unhealthy dietary and activity patterns potentially responsible for diseases linked to obesity (Hochberg, 2018; Speakman, 2013). To put the problem in perspective, the negative impact of “modernization” on health was mentioned in the Boston Medical and Surgical Journal over a hundred years ago, but the underlying drivers responsible for the deterioration of metabolic health may still be underappreciated (Jones et al., 2012).

Before the advent of modernization, humans derived inherent benefits from physical activity for 84,000 generations (O’Keefe et al., 2011). In less than 0.4% of that time period, humans rapidly accelerated into the current digital age (O’Keefe et al., 2011). Access to food has dramatically improved but unhealthy dietary and activity patterns have emerged (Pradhan, 2007), and we are now experiencing a worldwide obesity pandemic (Pontzer et al., 2012) responsible for almost 5 million premature deaths/year (Stanaway et al., 2018). Small populations of hunter‐gatherer societies remain well protected against chronic metabolic diseases as long as they do not adopt the patterns of a Westernized dietary and activity lifestyle (Pontzer et al., 2018). Movement constancy and the quality/quantity of nutrient intake seem to be largely responsible for their protective resilience (Kaplan et al., 2017; Liebert et al., 2013; Raichlen et al., 2017).

In our modern era, a tremendous amount of investigation has been focused on the use of complex dietary and exercise interventions to mitigate metabolic disease (Obesity, 2020). Despite considerable public interest in contemporary hunting and gathering, the acute influence of intensive wilderness hunting on metabolic health in the modern human is limited (Crittenden & Schnorr, 2017). While remote hunting expeditions in the 21st century do not replicate hunter‐gatherer societies, abrupt changes in physical activity and nutrient intake may foster beneficial adaptations. We are uniquely positioned to provide some investigative insight into these areas of interest, as numerous hunters pursue game in the unpredictable Alaskan environment with limited provisions and shelter (Walch et al., 2018).

In our original preliminary work (Coker et al., 2018), we described the total energy expenditure (TEE) and total energy intake (TEI) that led to negative energy balance during Alaska Backcountry Expeditionary Hunting (ABEH). The limited number of participants in the original study precluded our ability to evaluate changes in net energy balance, serum lipids, or variations in macronutrient intake, and did not include females. The objective of this study was to measure TEE, TEI, net energy balance, macronutrient intake, body composition, serum lipids, liver and metabolic panels, and intrahepatic lipid (IHL) in males and females during an 8–12 day ABEH immersion. We hypothesized that ABEH would promote beneficial changes in metabolic parameters (ie., body fat, serum lipids, and IHL) and maintain skeletal muscle despite negative energy balance and minimal protein intake in males and females.

## 2 METHODS

We recruited four men (age: 46 ± 7 year, BMI: 26 ± 1 kg/m^2^) and three women (age: 46 ± 11 year, BMI: 25 ± 3) for this study. After obtaining informed consent, all participants completed a comprehensive health history and were considered as healthy, nonsmoking participants. None of our participants were taking any medications nor were they symptomatic for cardiovascular, respiratory, neurological, or metabolic diseases. None had any chronic inflammatory conditions. All participants completed pre‐ and post‐ABEH visits that included: (a) measurement of body weight, body composition via dual‐energy x‐ray absorptiometry scans (General Electric iDXA; Madison, WI), and molecular imaging/spectroscopy (Toshiba Excelart/Vantage 1.5 T MRI/MRS, Canon, Õtawara, Tochigi, Japan) of muscle and liver, (b) blood sampling via LabCorp (1626, 30th Avenue, Fairbanks, AK), (c) measurement of TEE using the doubly labeled water (DLW) method (Schoeller, 1999), and d) assessment of TEI and macronutrient intake using written dietary and/or photographic records (Capling et al., 2017). All aspects of the study and related documentation were reviewed and approved by the University of Alaska Fairbanks (UAF) Institutional Review Board.

### 2.1 Alaska backcountry hunting immersion

All testing and examinations of our research participants were completed in the Clinical Research and Imaging Facility pre‐ and post‐ABEH immersion, and in the fall of 2018. These sessions were closely coordinated with Pristine Ventures and Shadow Aviation (Fairbanks, AK), minimizing the logistical burden on the participants, organizers, and remote bush pilots, while allowing us to perform the testing within 24 h of departure from and arrival in Fairbanks, AK. As our original manuscript indicated, hunters received basic instructions with regard to hunt preparation, meat preservation, and load carriage (Coker et al., 2018). We recognized the potential for shifts in background H_2_O isotope abundances in hunters originating from the contiguous states outside and those from Alaska. Therefore, subjects #1 and #2 were recruited from Alaska and the mid‐western United States, respectively, and received dummy, or sham doses of DLW. This allowed for correction for background shifts in isotope abundance.

### 2.2 Imaging measurements

Participants wore identical lightweight clothing or surgical scrubs during all body weight and imaging measurements. The participants were asked to lie motionless in the supine position during the iDXA measurements of body composition, including android and gynoid fat distribution. Android fat distribution refers to adipose tissue largely deposited in the trunk and upper body, while gynoid fat distribution is located in the hips, breasts, and thighs (Brody, 1999). The hands and arms were placed in a parallel position without touching the body, and within the required scan area. Feet were stabilized and movement was kept to a minimum through the use of a Velcro strap that also assisted with proper placement of the feet and legs. Calibration of the iDXA was performed at least three times/week.

We measured the cross‐sectional area of the upper thigh muscles (XT) and IHL using a Toshiba Excelart/Vantage 1.5T magnetic resonance imaging/magnetic resonance spectroscopy (MRI/MRS) system (Canon, Ōtawara, Tochigi, Japan) as previously described (Coker et al., 2018). Acquisition of axial and coronal T1‐weighted images was collected using a Field Echo sequence (TR = 172 172 msec, TE = 90 msec). Axial T2 images were acquired using a Fast Spin Echo sequence (TR = 3700 msec, TE = 90 msec). One technician selected seven of the axial T1‐weighted images based on the identification of the largest clearly visible scan in the belly of the thigh muscle. Six progressive images were chosen by moving distally toward the patella. Subsequently, all seven XT images were analyzed by the same technician using OsiriX software (Pixmeo, Bernex; Hulmi et al., 2009). Raw data from the spectra for determination of IHL, including an un‐suppressed water reference, were converted to ascii format using a custom script before analysis using the jMRUI software. All spectra were Fourier transformed, phased, and referenced (1.4 ppm for lipid spectra, 4.8 ppm for water reference). The signals were fit using the AMARES non‐linear‐least‐squares algorithm within jMRUI. The results from both the lipid spectra and water reference spectra were then used to calculate a lipid‐to‐water ratio (Bennett et al., 2012).

### 2.3 Isotopic methodology

A baseline urine sample was collected at 22:00 hr on the evening prior to departure into the remote backcountry. Immediately afterward, all participants received either an oral “sham” dose or an oral dose of DLW. Two of the seven participants originated from the contiguous United States, supporting the rationale for the need of a sham dose to would allow for adjustments of potential alterations in background enrichments of ^18^O and ^2^H (Schoeller et al., 1986). While in the field for 8–12 days, all participants collected at least three urine samples as previously described (Coker et al., 2018). This dosing and urine sampling protocol has been previously established (Coker et al., 2018; Schoeller et al., 1986).

Participants received detailed instructions for the collection and placement of their own urine samples into sterile, polypropylene, nonpyrogenic, RNase/DNase‐free tubes (Corning, Inc.) to be wrapped in Parafilm^TM^ (Bernis NA). All samples were kept according to directions, separated from other participants, in dry storage, and at an ambient temperature of 4° to 10°C. Upon the participants’ return to Fairbanks, AK, all samples were immediately frozen.

The analysis of isotopic abundance was completed at the Isotope Ratio Core Laboratory at the University of Wisconsin, Madison, WI (Hoyt et al., 1991). We fitted the absolute value of an exponential model to preclude any undesirable influence of variations in background abundance, as previously described (Coker et al., 2018). We then used the theoretical constructs established by Stroud et al. (1993), basing the extrapolated infinite time background estimate on average from the exponential fit of abundance versus time in the participants receiving the sham doses. Production of CO_2_ was calculated as previously described and utilized a respiratory exchange ratio of 0.78 for the calculation of total energy expenditure for the period in the field beginning with the first urine collected at the hunt site and ending upon return to Fairbanks, AK (Thorsen et al., 2011).

### 2.4 Dietary intake

We requested that participants to keep a daily record of all food and drink intake, along with caloric content consumed during the ABEH. All participants collected these food diaries based on our verbal and written instructions and were collected immediately upon the participants return from the ABEH. We also recorded the exact amount and type of food that each participant took into the field. With the exception of animals harvested and ingested (also recorded) on the ABEH, there were no other sources of food available. The energy and macronutrients of moose and caribou were extracted from SELFNutritionData weblink (SELFNutritionData, 2020). This combined strategy of food inventories, in conjunction with written and photographic dietary records, allowed us to precisely determine the macronutrient and energy content of all foods consumed.

### 2.5 Statistical analysis

We analyzed our data using a combination of Microsoft Excel, iDXA Encore, Osirix, and Prism 9 software. Since two participants received “sham” doses of DLW to control for shifts in background enrichment, data presented on TEE and TEI include three male and two female participants. All other data include seven total participants. We used paired *t*‐tests to evaluate alterations in pre‐ABEH and post‐ABEH data. Linear regression analysis was used to determine the relationship between TEE and lean tissue mass (LTM). Statistics were considered significant with a *P*‐value of less than 0.05. Data are presented as means ± SD.

## 3 RESULTS

### 3.1 ABEH overview

Three (#5, #6 and #7) of the seven participants were successful in harvesting an animal (one caribou and two moose, respectively). Two (#3 and #4) participants were mountain hunting for sheep (SHP) and five participants (#1, #2, #5, #6, and #7) were “float‐dragging” a raft through a remote river corridor for moose and caribou (FD) (Bartlett, 2014).

### 3.2 Total energy expenditure

**FIGURE 1:**
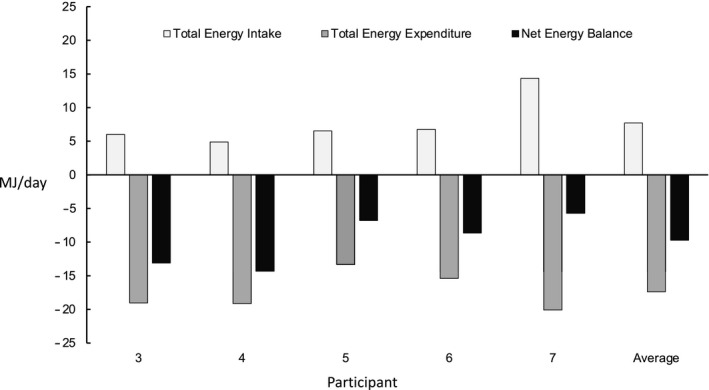
Total energy intake, total energy expenditure and net energy balance in participants #3, #4, #5, #6, #7 and their average values. Participants #1 and #2 received sham doses of DLW

**FIGURE 2:**
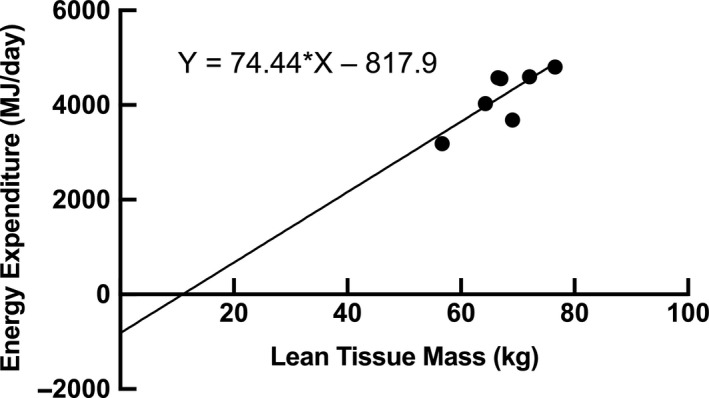
Relationship between total energy expenditure (TEE) and lean tissue mass (LTM) in all participants (*p* = 0.03)

The average TEE was 17.4 ± 2.6 MJ/day with 19.1 ± 0.1 MJ/day and 16.3 ± 2.4 MJ/day for SHP and FD hunters, respectively. The average absolute TEE was 18.2 ± 2.0 MJ/day and 16.2 ± 2.9 MJ/day for males and females, respectively (Figure 1). When expressed relative to pre‐ABEH body composition measurements, TEE/LTM was similar in males (60.6 ± 4.3 MJ·kg LTM^−1^·day^−1^) and females (64.3 ± 5.8 MJ·kg LTM^−1^·day^−1^; Figure 1). There was a significant relationship between TEE and LTM (Y = 74.44*X − 817.9; *p* = 0.03; Figure 2).

### 3.3 Total energy intake

The TEI was 7.7 ± 3.4 MJ/day with 5.4 ± 1.9 MJ/day and 9.4 ± 4.0 MJ/day for SHP and FD hunters, respectively. Males and females consumed a TEE of 9.4 ± 4.2 MJ/day and 5.8 ± 4.2 MJ/day, respectively. Based on an average TEE of 16.3 MJ/day and a TEI of 7.7 MJ/day, the net energy balance was −9.7 MJ/day, or −2320 calories/day (Figure 1).

### 3.4 Macronutrient intake

**FIGURE 3:**
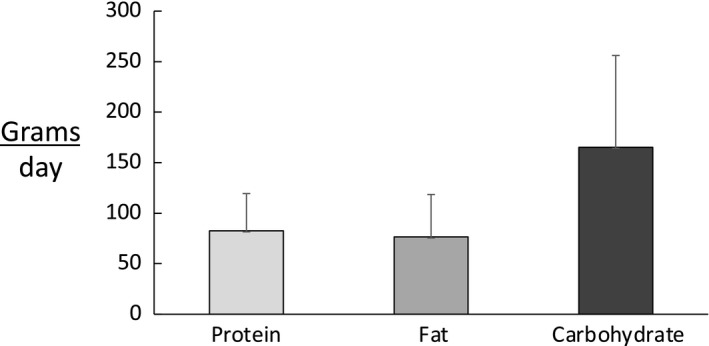
Average dietary intake of protein, fat and carbohydrate in grams/day in all participants

The average intake of protein, fat, and carbohydrates was 84 ± 37 g/day, 77 ± 42 g/day, and 165 ± 91 g/day, respectively (Figure 3). On the percentage of caloric intake, protein, fat, and carbohydrate was 20 ± 9%, 41 ± 22%, and 39 ± 22%, respectively. The average protein intake relative to body weight was 1.03 ± 0.40 g/kg body weight in the five participants whose TEE was 17.4 ± 2.1 MJ/day and TEI was 7.7 ± 3.4 MJ/day. The average protein intake in all seven participants was 1.01 ± 0.11 grams/kg body weight; highlighting similar levels of protein intake among the two participants who received sham doses of DLW for determination of potential changes in background enrichment.

### 3.5 Body weight and body composition

There were significant reductions in body weight (*p* = 0.01) and BMI (*p* = 0.01; Table 1). Total fat mass, percent fat, android fat, arm fat, and trunk fat were reduced. There were strong trends toward the reduction in leg fat (*p* = 0.06) and gynoid fat (*p* = 0.06). The android/gynoid ratio was reduced (*p* = 0.005) along with decreased visceral fat mass (*p* = 0.006), and visceral fat volume (*p* = 0.006); representing the most remarkable alterations in body fat (Table 2).

**TABLE 1:** Clinical characteristics (*n* = 7)

	Pre‐ABEH	Post‐ABEH
Age	46 ± 7	
Height (m)	1.8/0.2	
Weight (kg)	81.8 ± 10.2	80.3 ± 9.7*
BMI (kg/m^2^)	25.7 ± 2.2	25.4 ± 2.3*

Note

Data are presented as Mean ± SD.

* Denotes significant difference between pre‐ and post‐ABEH.

**TABLE 2:** Adipose parameters

	Pre‐ABEH	Post‐ABEH
Body fat (%)	24.9 ± 5.9	23.0 ± 6.5
Arm fat (%)	20.6 ± 6.3	19.6 ± 6.1*
Leg fat (%)	22.0 ± 7.8	21.2 ± 9.7^^^
Trunk fat (%)	26.7 ± 6.1	23.8 ± 7.2*
Android fat (%)	28.0 ± 7.2	24.2 ± 8.1*
Gynoid fat (%)	25.7 ± 7.7	24.4 ± 8.0*
Total fat (kg)	18.4 ± 3.6	16.7 ± 4.3*
Arm fat (kg)	1.0 ± 0.1	0.9 ± 0.1*
Leg fat (kg)	2.6 ± 0.8	2.5 ± 0.8^^^
Trunk fat (kg)	10.0 ± 2.6	8.7 ± 2.9*
Android fat (kg)	1.5 ± 0.4	1.2 ± 0.4*
Gynoid fat (kg)	2.9 ± 0.8	2.8 ± 0.9^^^
Visceral fat mass (kg)	0.61 ± 0.26	0.50 ± 0.21*
Visceral fat volume (cm^2^)	680 ± 271	535 ± 219*

* Denotes significant difference (*p* < 0.05) between pre‐ and post‐ABEH.

^^^ Denotes trend toward a difference (*p* < 0.10) between pre‐ and post‐ABEH. Data are presented as Mean ± SD.

There were no significant changes in whole body LTM, arm LTM, leg LTM, android LTM, or gynoid LTM. A strong trend (*p* = 0.12) toward an increase in trunk LTM was demonstrated. A significant increase in trunk lean mass (*p* = 0.04) and total LTM was noted in women (*p* = 0.01; Table 3). There was a strong trend (*p* = 0.06) toward a relationship between protein intake and ABEH‐induced changes in LTM (*p* = 0.09; Figure 3). In fact, all participants gained between 0.2 and 1.4 kg of LTM except for one individual who consumed 0.5 g/kg of protein and lost 2.0 kg of LTM. (Figure 3). There were no significant changes in XT (Table 3).

**TABLE 3:** Musculoskeletal parameters

	Pre‐ABEH	Post‐ABEH
Total lean mass (kg)	59.0 ± 10.4	59.6 ± 10.1
Arm lean mass (kg)	7.84 ± 2.05	7.86 ± 1.96
Leg lean mass (kg)	20.0 ± 3.7	20.0 ± 3.8
Cross sectional area thigh (cm^2^)	148.6 ± 25.6	146.9 ± 21.6
Trunk lean mass (kg)	27.6 ± 4.5	28.1 ± 4.3^^^
Android lean mass (kg)	3.87 ± 0.73	3.94 ± 0.75*
Gynoid lean mass (kg)	8.95 ± 1.52	9.01 ± 1.50
Arm bone mineral content (kg)	0.50 ± 0.11	0.50 ± 0.11
Leg bone mineral content (kg)	1.22 ± 0.21	1.23 ± 0.22*
Trunk bone mineral content (kg)	0.92 ± 0.15	0.93 ± 0.14
Android bone mineral content (kg)	0.59 ± 0.14	0.59 ± 0.13
Gynoid bone mineral content (kg)	0.32 ± 0.06	0.33 ± 0.06*
Total bone mineral content (kg)	3.24 ± 0.49	3.25 ± 0.48

* Denotes significant difference (*p* < 0.05) between pre‐ and post‐ABEH.

^^^ Denotes trend toward a difference (*p* < 0.10) between pre‐ and post‐ABEH. Data are presented as Mean ± SD.

No significant changes in arm, trunk, android, or total bone mineral content were indicated. On the other hand, increases in bone mineral content were detected in the leg (*p* = 0.04) and gynoid (*p* = 0.03) regions (Table 3).

When categorized by males and females, body mass, body mass index, fat mass, and whole body LTM was 90 ± 4 kg and 72 ± 8 kg, 26 ± 1 kg/m^2^ and 25 ± 3 kg/m^2^, 19 ± 3 kg, and 19 ± 4 kg, and 66 ± 3 kg and 49 ± 8 kg, respectively.

### 3.6 Intrahepatic lipid

**FIGURE 4:**
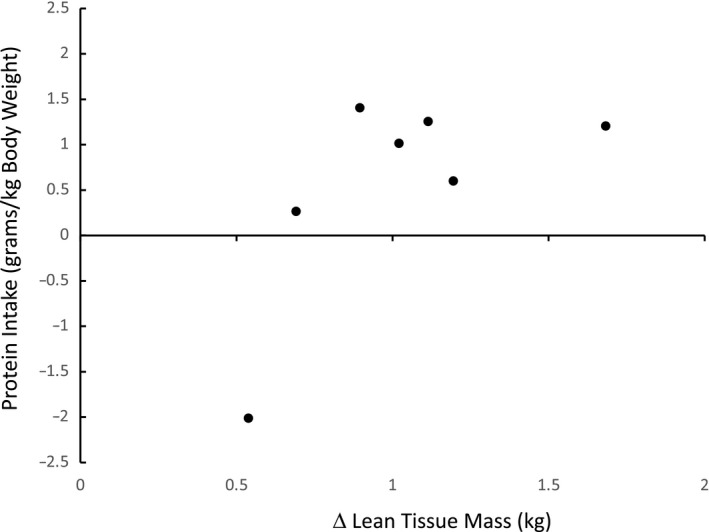
Relationship between average protein intake and change in lean tissue mass (LTM) in all participants (*p* = 0.09)

There was a significant reduction in MRI/MRS‐derived IHL (*p* = 0.007; Figure 4). This reduction in IHL was consistent with our previous study (Kaplan et al., 2017), but these data now include females.

### 3.7 Blood/Serum parameters

No significant changes were noted in the basic metabolic panel (i.e., glucose, blood urea nitrogen (BUN), creatinine, estimated glomerular filtration rate, BUN/creatinine, sodium, potassium, chloride, carbon dioxide, and calcium; Table 4). All values were within normal limits. With regard to the lipid panel, there were significant reductions in total cholesterol (*p* = 0.01), LDL‐cholesterol (*p* = 0.004), VLDL‐cholesterol (*p* = 0.02), and triglycerides (*p* = 0.02; Table 4). While HDL‐cholesterol did not decrease (*p* = 0.18), there was a reduction in triglyceride/HDL‐cholesterol (*p* = 0.02). Significant reductions in total protein (*p* = 0.007), albumin (*p* = 0.01), and total bilirubin (*p* = 0.01) were noted (Table 4). Overall, there were no changes in alanine aminotransferase, alkaline phosphatase, and aspartate aminotransferase, but each one was significantly reduced in females (*p* = 0.004, 0.004, and 0.02, respectively; Table 4).

**TABLE 4:** Blood parameters

	Pre‐ABEH	Post‐ABEH
*Metabolic Panel*		
Glucose (mg/dl)	97.5 ± 19.1	89.8 ± 15.8
Blood urea nitrogen (mg/dl)	17.3 ± 3.7	15.5 ± 2.6
Creatinine (mg/dl)	1.0 ± 0.1	1.0 ± 0.1
Estimated glomerular filtration rate (mL/min/1.73)	73.2 ± 8.2	75.0 ± 11.5
Blood urea nitrogen/creatinine	17.2 ± 4.3	15.5 ± 2.8
Sodium (mmol/L)	140.0 ± 0.9	140.3 ± 1.8
Potassium (mmol/L)	4.2 ± 0.1	4.3 ± 0.2^^^
Chloride (mmol/L)	100.5 ± 2.0	101.5 ± 1.0^^^
Carbon dioxide (mmol/L)	23.7 ± 1.6	24.2 ± 1.8
Calcium (mg/dl)	9.6 ± 0.2	9.4 ± 0.2*
*Lipid Panel*		
Triglyceride (mg/dl)	92.5 ± 40.8	57.8 ± 25.1*
Total cholesterol (mg/dl)	196.7 ± 31.4	153.2 ± 43.1*
LDL‐cholesterol (mg/dl)	105.7 ± 33.0	80.8 ± 27.2*
VLDL‐cholesterol (mg/dl)	18.7 ± 8.1	11.5 ± 5.1*
HDL‐cholesterol (mg/dl)	72.3 ± 20.3	77.5 ± 30.8
Triglyceride/HDL‐cholesterol	1.4 ± 0.9	0.9 ± 0.5*
*Hepatic function panel*		
Total protein (g/dl)	7.1 ± 0.2	6.7 ± 0.2*
Albumin (g/dl)	4.6 ± 0.1	4.4 ± 0.2*
Bilirubin (g/dl)	0.45 ± 0.14	0.52 ± 0.16*
Alanine aminotransferase (IU/L)	20.7 ± 1.6	31.8 ± 8.2*
Alkaline phosphatase (IU/L)	50.2 ± 11.5	51.0 ± 7.5
Aspartate aminotransferase (IU/L)	38.0 ± 15.5	38.8 ± 12.6

* Denotes significant difference (*p* < 0.05) between pre‐ and post‐ABEH.

^^^ Denotes trend toward a difference (*p* < 0.10) between pre‐ and post‐ABEH. Data are presented as Mean ± SD.

## 4 DISCUSSION

The results of this study demonstrated high levels of TEE and modest TEI that consistently contributed to negative energy balance during the 8–12 day ABEH in healthy males and females. The short‐term period of negative energy balance elicited reductions in body weight and adipose tissue. With the percentage of dietary protein, fat, and carbohydrate intake at ~20%, 40%, and 40%, respectively, LTM and XT were preserved in spite of the arduous physiological and field conditions. We also demonstrated rapid, significant reductions in serum lipids and IHL that met or exceeded dietary, exercise, and/or pharmaceutical interventions over longer periods of time in individuals at a greater risk for metabolic diseases (Hays et al., 2008).

Exercise training interventions have demonstrated reductions in body weight, adipose tissue, and visceral fat (Da Silva et al., 2020). In efforts to improve outcomes in a short period of time, high‐intensity interval training has also been utilized successfully to promote beneficial alterations in body weight, especially when combined with resistance training (Stoner et al., 2016). Expedient alterations in body composition in this particular study were likely linked to high levels of negative energy balance (i.e., −9.7 MJ/day) elicited by sustained elevations in physical activity (Strasser et al., 2007). That being said, long‐term weight loss remains difficult to achieve for many individuals (Swift et al., 2014), and this may be due to the inability to maintain sufficient activity.

The minimum recommendations of 150 min/week of moderate or 75 min/week of vigorous physical activity are generally insufficient to promote clinically significant weight loss (Donnelly et al., 2009). The exercise duration threshold seems to occur at ~225 min/week with interventions lasting at least 12 weeks or longer in obese individuals (Donnelly et al., 2009). The ABEH immersion provoked a swift decline in body weight and fat compared to much longer supervised training regimens. If we were to extrapolate the average negative energy balance of the entire ABEH immersion, it would be equivalent to ~80 MJ in only the hunters who received DLW. Theoretically, the negative energy balance would have contributed to the loss of ~2.5 kg of body weight. We measured a reduction in 1.5 kg of body weight and 1.7 kg of fat loss in these same individuals, with almost 80% of the fat reduction occurring in the trunk region of all seven participants. The ABEH immersion promoted a rapid reduction in trunk and visceral fat, potentially offering unique benefits in the protection against metabolic risk in a middle‐aged cohort of participants as recently outlined by Barbour‐Tuck et al. (2019).

In our prior work, we were not able to adequately evaluate ABEH‐induced alterations in serum lipids (Coker et al., 2018). We now describe consistent reductions in total cholesterol, LDL‐cholesterol, VLDL‐cholesterol, triglycerides, and triglyceride/HDL‐cholesterol in healthy females and males whose initial lipid levels were already within normal limits. The clinical significance of these data is based on the abundant evidence that supports the link between elevated atherogenic lipids and cardiovascular disease (MacMahon et al., 2007). Due to the deleterious influence of elevated circulating lipids on cardiovascular health, studies have employed exercise training paradigms to improve lipid profiles with variable results (Pedersen & Saltin, 2006). In a recent seminal review article by Leon and Sanchez (2001) that examined randomized controlled trials in individuals with a slightly higher BMI than our own cohort of participants, it was concluded that the responsiveness of blood lipids to exercise training programs of 11 weeks to 1 year were inconsistent at best. In fact, only three of the 10 trials described a significant reduction in LDL‐cholesterol or triglycerides.

A number of variables can affect serum lipids, such as the lack of laboratory standardization (Leon & Sanchez, 2001), genetic variations (Despres et al., 1988), and exercise training that does not result in significant weight loss (Wang & Xu, 2017). It has been proposed that for every kg of body weight loss, there will be a 0.8 mg/dl reduction in LDL‐cholesterol (Goldberg et al., 2011; Kodama et al., 2007). Using this calculation, we would have estimated a 1.2 mg/dl reduction in LDL‐cholesterol. Instead, we reported a 24.9 mg/dl reduction in LDL‐cholesterol. Given the well‐described sensitivity of HDL‐cholesterol (Chapman et al., 2011), it was somewhat surprising that HDL‐cholesterol did not change. However, the triglyceride/HDL‐cholesterol ratio, a strong predictor of all‐cause mortality linked to cardiovascular disease (Hamaguchi et al., 2007) was reduced, making the rapid improvements in IHL all the more intriguing. It is very well accepted that the development of nonalcoholic fatty liver disease represents a hepatic manifestation of metabolic disease that has been linked to an increased risk of atherosclerotic cardiovascular disease (Donnelly et al., 2005). Excessive positive energy balance combined with (a) triglycerides derived from hepatic de novo lipogenesis, (b) fatty acids derived from stored fat, and (c) triglyceride rich lipoproteins, overwhelm normal lipid flux and hepatic function (Shojaee‐Moradie et al., 2016). In the presence of a relatively well‐balanced diet with respect to protein, fat, and carbohydrate intake, exercise may protect against these etiological processes through a reduction in hepatic *de novo* lipogenesis affecting lipid flux via an increase in the VLDL_1_‐triglyceride fractional catabolic rate (Abadi et al., 2009). The inherent movement constancy of the 8‐ to 12‐day ABEH immersion promoted improvements in serum lipids and IHL in generally healthy individuals. Based on the complex molecular regulatory factors that have independent effects on cardio‐metabolic health (Ryu et al., 2015), these results suggest the efficacious synergism between an overall reduction in sedentary time (Johnson et al., 2009), mild to intense exercise (Thoma et al., 2012), and weight loss (Coker & Wolfe, 2018).

The atrophy of skeletal muscle during weight loss has been studied extensively in a wide range of individuals due to its inextricable link to deleterious alterations in physical function and/or performance (Cava et al., 2017; Church et al., 2019; Palus et al., 2014; Tassone & Baker, 2017). Our ABEH immersion resulted in negative energy balance largely due to the influence of sustained physical activity on TEE, coupled with the various barriers to eating enough to maintain energy balance. These challenges may manifest themselves through inherent difficulties of nutrient access during intense movement, additional load carriage, and/or insufficient time or energy to prepare food. Given the remote environment, self‐reliance of participants, and difficult terrain embedded into the ABEH, these conditions may be somewhat similar to military training exercises, albeit not absolutely equivocal (Alemany et al., 2008). The level of TEE and TEI in our study was remarkably analogous to previous investigations that described changes in body composition during military exercises of a similar duration (Margolis et al., 2014). Whereas LTM was reduced in both of these prior studies, a similar reduction was noted with one individual in the ABEH with low protein intake in this study. Muscle loss may also be influenced by sleep deprivation commonly experienced in actual military operations (O’Hara et al., 2014). On the other hand, studies in our laboratory have demonstrated the retention of LTM despite high levels of TEE further complicated by sleep deprivation under challenging environmental conditions (Johannsen et al., 2018; Schalt et al., 2018), but their interpretation has been limited due to the lack of data on dietary macronutrient intake.

We report no reductions in LTM or MRI‐derived skeletal muscle (i.e., XT) in this study, with an average protein intake of 1.0 ± 0.1 g/kg and average macronutrient intake slightly lower than the recommendations provided to individuals engaged in a general fitness program (Hart et al., 2017; Kreider et al., 2010). In fact, LTM in the android region and bone mineral content in the leg and gynoid region were increased, potentially linked to the overload of movement constancy combined with variable amounts of load carriage on posture and stability (Pasiakos & Margolis, 2017). One of our participants who was sheep hunting did experience the loss of total LTM (−2.4 kg), trunk LTM (−1.5 kg), leg LTM (−0.9), and XT (i.e., −15.7 cm^2^), with a corresponding lower dietary protein intake of 0.54 g/kg of body weight (Figure 3). Longer term participation in ABEH or similar scenarios could also eventually put LTM and XT at a greater risk (Tassone & Baker, 2017). On the other hand, short‐term exposure to ABEH may require strategies that provide the minimal amount of nutrient delivery needed to maintain skeletal muscle, instead of dietary recommendations more relevant in the context of supervised, periodized training (Fallon et al., 1999).

The high physiological stress of prolonged physical activity, further complicated by episodes of heavy load carriage, affects muscle and hepatic metabolism (Kupchak et al., 2014). Thus, it was not entirely surprising that total protein, albumin, bilirubin, and alanine amino transferase were affected by the ABEH immersion. Runners competing in the 161 km Western States Endurance Run, a more acutely stressful event that the ABEH, exhibited similar baseline total protein and albumin levels to our participants, and these values decreased similarly during the first half of the event (Nagel et al., 1990). The increase in bilirubin in this study was also consistent with ultra‐marathon running and associated with hemolysis (Shin et al., 2016). Alanine transaminase was also increased, indicative of hepatic stress (Blonde et al., 2018). While none of the parameters exceeded or fell below normal limits, alterations in the parameters of the hepatic panel provide solid evidence of the elevated physiological stress that occurred during the ABEH (Shin et al., 2016).

We recognize the limitations of an unscripted event that lack internal validity with regard to control for environmental conditions, sleep, exercise intensity, and duration, and small differences in the lengths of the ABEH excursion itself. With the incredible time and resources (i.e., bush travel, logistical planning, satellite communication) required for a field study needing extensive remote support, we also realize that our small sample size reduced our statistical power. On the other hand, it would be fundamentally impossible to replicate these unscripted field‐based activities in the form of a laboratory‐based study due to day to day alterations in weather, variable terrain, tasks at hand, and amount of movement constancy required throughout each day. Even if this type of study was attempted in the laboratory, it would be unlikely to capture the external validity of the data presented using our ABEH approach (Blonde et al., 2018). Making some sex‐based comparisons would be desirable. Future studies have already been planned to evaluate the potential for sex differences in the metabolic response to the ABEH scenario.

## 5 CONCLUSION

We have presented compelling evidence that promotes the efficacy of ABEH on improvements in biomarkers typically associated with an increased risk for metabolic disease in females and males. Even in individuals who were generally healthy, 8–12 days of unscripted activity without any attempt to control or manipulate dietary intake was sufficient to promote rapid improvements in serum lipids and IHL. We recognize that the recommendations for protein intake (i.e., 1.4–2.0 g/kg/day) and carbohydrate (i.e., 3–5 g/kg/day) provided by the International Society of Sports Nutrition may be considerably higher during the macrocycle of a training period or a competitive scenario for an athlete (Carbone and Pasiakos, 2019; Hector & Phillips, 2018; Phillips et al., 2016). However, the practical reality of these dietary recommendations must be balanced against the need for agile operations over an 8‐ to 12‐day period at a relatively constant exercise intensity, especially when carrying all provisions in a backpack across difficult terrain or a raft being moved over many kilometers of a dry creek bed. We suggest that the minimal dietary protein of 0.8 g/kg/day under these types of unscripted and self‐reliant conditions is just that; the absolute minimal amount of protein potentially required to maintain skeletal muscle in the context of an anabolic stimulus provided by physical activity (Devlin et al., 1990; Williams et al., 1996).

## 6 ACKNOWLEDGMENTS

We express our sincere appreciation to our participants that volunteered their time and effort for this study. We also thank Jack Corbett for his technical assistance in this study.

## CONFLICT OF INTEREST

No conflicts of interest, financial or otherwise, are declared by the authors.

## AUTHORS’ CONTRIBUTIONS

M.S.C., B.C.R., L.B., and R.H.C. conceived and designed the study; M.S.C., C.J.M., T.B., and R.H.C. performed the experiments; M.S.C., T.C.S., D.A.S., B.R.N., and R.H.C. analyzed the data, M.S.C., K.R.L., T.C.S., D.A.S., and R.H.C. interpreted the results of experiments; M.S.C., B.C.R., L.B., and R.H.C. prepared tables and figures; M.S.C. and R.H.C. drafted the manuscript; M.S.C., K.R.L., C.J.M., B.C.R., T.C.S., D.A.S., B.R.N., T.B., L.B. and R.H.C. edited and revised the manuscript; M.S.C., K.R.L., C.J.M., B.C.R., T.C.S., D.A.S., B.R.N., T.B., L.B., and R.H.C. approved the final version of manuscript.

7

**FIGURE 5:**
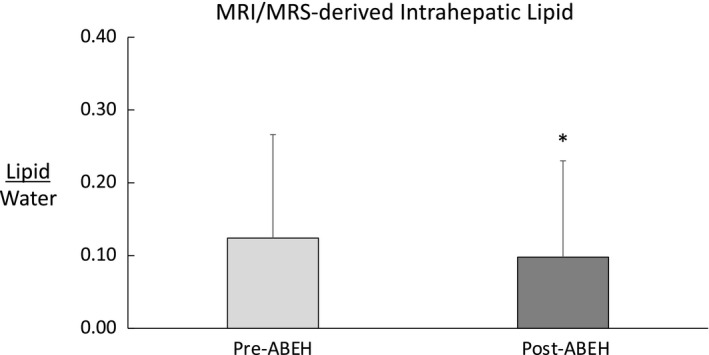
Intrahepatic lipid at pre‐ABEH and post‐ABEH in all seven participants. *Represents statistically significant difference (*p* = 0.02)
